# Distribution of Diagnoses and Clinical and Imaging Characteristics in 1,322 Consecutive Suspected Stroke Patients

**DOI:** 10.3389/fneur.2021.753183

**Published:** 2021-10-20

**Authors:** Peter B. Sporns, Alex Brehm, Caroline Hilgers, Nikolaos Ntoulias, Ioannis Tsogkas, Marios Psychogios

**Affiliations:** ^1^Department of Neuroradiology, Clinic of Radiology and Nuclear Medicine, University Hospital Basel, Basel, Switzerland; ^2^Department of Diagnostic and Interventional Neuroradiology, University Medical Center Hamburg-Eppendorf, Hamburg, Germany; ^3^Department of Neuroradiology, University Medical Center Göttingen, Göttingen, Germany; ^4^Department of Medicine, University of Patras, Patras, Greece

**Keywords:** stroke, endovascular treatment, resources, intravenous thrombolysis, mechanical thrombectomy

## Abstract

**Background:** Endovascular thrombectomy (EVT) has become the standard of care for large-vessel occlusion strokes, but several barriers for implementing an optimal organization of stroke management remain. Major issues include the lack of reliable data on the percentage of stroke patients potentially eligible for EVT especially in times of expanding indications for EVT. Our aim was therefore to study the frequencies of possible EVT-eligible patients such as patients with medium-vessel occlusions, patients with low Alberta Stroke Program Early Computed Tomography Scores (ASPECTS), patients presenting in an extended time window after onset of symptoms, and patients with mild symptoms at presentation (National Institutes of Health Stroke Scale, NIHSS ≤ 5). We also give detailed imaging and clinical information about the patients presenting with intracranial hemorrhage and other ischemic stroke mimics stratified by symptoms at presentation.

**Methods:** Cohort study of all consecutive patients with suspected acute stroke presenting to a tertiary care center in Germany between September 1, 2016, and August 31, 2017. Baseline and follow-up clinical and imaging characteristics were collected from patients' medical charts.

**Results:** Of 1,322 patients with a suspected acute stroke, 592 (44.8%) had ischemic strokes, 221 (16.7%) had hemorrhagic strokes, 190 (10.9%) had transient ischemic attacks (TIAs), and 319 (24.1%) were classified as stroke mimics. Stroke severity was mild (NIHSS ≤ 5) in 866 (65.5%) patients; 15.7% of the patients with an occlusion of the anterior circulation had an ASPECTS ≤ 5, 17.4% of the patients with an ischemic stroke had distal vessel occlusions, and 49% of the patients presented later than 6 h after onset of symptoms.

**Conclusion:** Our results help to plan resources in thrombectomy-capable centers in times of expanding indications for EVT where resources will have to be adjusted to patients with low-NIHSS, low-ASPECTS, and distal occlusions, and patients presenting in the extended time window, which may altogether account for an additional 20% of all ischemic stroke patients.

## Introduction

Endovascular thrombectomy (EVT) has become the standard of care for large-vessel occlusion strokes, and acute management of ischemic stroke has dramatically changed since the demonstration of the efficacy of EVT (1). However, several barriers for implementing an optimal organization of stroke management remain. A major issue among them is the lack of reliable data on the percentage of stroke patients eligible for EVT and their characteristics. Even though the number of patients with large-vessel occlusions and occlusions of the M2 segment of the middle cerebral artery has been estimated (2–5), detailed information about further characteristics such as frequency of stroke mimics and intracranial hemorrhages is lacking. Moreover, the available information is mostly confined to large-vessel occlusion strokes, but in times of expanding indications for EVT (6), the frequency of more medium-vessel occlusions, the distribution of Alberta Stroke Program Early Computed Tomography Score (ASPECTS), the time window of presentation, and the National Institutes of Health Stroke Scale (NIHSS) of those patients are also of crucial importance for planning thrombectomy resources. We therefore conducted a cohort study of consecutive patients admitted with suspected stroke to a tertiary care hospital and describe all these strategically important variables. We also give detailed imaging and clinical information about the patients presenting with intracranial hemorrhage and other ischemic stroke mimics and stratify the results by NIHSS at presentation.

## Methods

The authors declare that the underlying data will be made available upon reasonable request by the corresponding author.

### Study Population

This study includes a prospectively collected cohort of all consecutive patients with suspected acute stroke presenting to a tertiary care center in Germany between September 1, 2016, and August 31, 2017. Acute stroke was suspected if patients (1) were transported to the hospital from an ambulance under the “stroke code (high probability of stroke according to the emergency doctor or paramedic),” (2) presented independently to the hospital and were triaged as suspected acute stroke after the first contact, or (3) were transferred from another hospital either with a confirmed stroke or suspected stroke. Patients were identified by screening reports of all head CTs performed in the study period and were validated by checking neurological clinical reports. All acute patients with suspected acute stroke undergo emergent CT at our center. Demographic information and risk factors were collected from the hospital information system. The National Institute of Health Stroke Scale (NIHSS) is routinely collected by a certified stroke neurologist on admission and discharge. The final diagnosis and modified Rankin Scale Score at discharge (mRS) were obtained from the final clinical report. Imaging findings were obtained through reviewing all scans—occlusion location was rated on baseline CT angiography (CTA) and validated on the Digital Subtraction Angiography (DSA) images if available. The Alberta Stroke Program Early CT Score (ASPECTS) was only evaluated in ischemic stroke patients with an occlusion of the anterior circulation. Additionally, the ABCD2 score estimating the risk of stroke after transient ischemic attack and minor stroke was calculated (7). Medium-vessel occlusions were defined as an occlusion visible on the CTA other than the M1, ICA, or BA. Small vessel occlusions were defined as an acute ischemic stroke (as determined by the non-contrast CT or perfusion CT) with no visible occlusion on the CTA. The local ethics committee waived the need for a formal application or a separate consent concerning the inclusion in our observational database.

### Hospital Setting

The hospital has 1,563 beds and has 55,159 inpatient and 222,303 outpatient cases per year. The Department of Neurology has 4,791 inpatient cases per year (as per 2017). It serves as the primary tertiary hospital for eight German districts and has a catchment area of roughly 1,100,000 persons and 6,500 km^2^. The largest city within its catchment area has 118,911 inhabitants, and the region has a below average population density. It is part of a stroke network with an integrated imaging service and serves as a referral center for 17 primary hospitals.

### Statistical Analyses

Statistical analysis was performed using GraphPad Prism 9 (GraphPad Software, San Diego, CA, USA, https://www.graphpad.com/, 2021). Parametric variables are stated as mean ± standard deviation (SD). Non-parametric or ordinary variables are presented as median and interquartile range (IQR). No interference statistics were performed. All data are presented for the whole collective, patients with a mild stroke (NIHSS ≤ 5), a moderate stroke (NIHSS 6–12) and for patients with a severe stroke (NIHSS > 12).

## Results

In the 1-year study period, 1,322 patients presented with a suspected acute stroke, of whom 592 (44.8%) had ischemic strokes, 110 (8.3%) had intraparenchymal hemorrhages (ICHs), 90 (6.8%) had subarachnoid hemorrhages (SAHs), 21 (1.6%) had subdural hematomas (SDHs), 190 (14.4%) had transient ischemic attacks (TIAs), and 319 (24.1%) were classified as stroke mimics. Mean age at admission was 70.8 years [standard deviation (SD) ±14.9 years], 385 (29.1%) were more than 80 years old, and 605 (45.8%) were female. Stroke severity was mild (NIHSS ≤ 5) in 866 (65.5%), moderate (NIHSS 6–12) in 282 (21.3%), and severe (NIHSS ≥ 13) in 174 (13.2%) patients. Arterial imaging was performed in 837 patients (63.3%), and of those 296 (22.4%) patients who received additional CT perfusion (CTP). Overall, 135 (10.2%) patients underwent EVT and 167 (12.6%) of the patients were treated with intravenous tissue plasminogen activator (i.v.-tPA). In total, 675 patients (51.1%) presented within 6 h of symptom onset, 172 patients (13%) between 6 and 12 h of onset, 278 patients (21%) between 12 and 24 h of onset, and 197 patients (14.9%) even later than 24 h after onset of symptoms. Detailed information on baseline and imaging characteristics is presented in Table 1.

**Table 1:** Baseline characteristics.

**Variable**	**All patients (*n* = 1,322)**	**NIHSS ≤ 5 (*n* = 866, 65.5%)**	**NIHSS 6–12 (*n* = 282, 21.3%)**	**NIHSS ≥ 13 (*n* = 174, 13.2%)**
Female, *n* (%)	605 (45.8%)	378 (43.6%)	136 (48.2%)	91 (52%)
Age, Mean (SD)	70.8 (±14.9)	69.1 (±15.3)	73.5 (±13.8)	74.8 (±12.7)
Over 80 years old	385 (29.1%)	222 (25.6%)	99 (35.1%)	64 (36.8%)
NIHSS, median (IQR)	4 (2-8)	2 (2-4)	9 (8-11)	17 (4-21)
Transfer patients	240 (18.2%)	110 (12.7%)	62 (22.0%)	68 (39.1%)
**First diagnostic step**				
Native CT	485 (36.7%)	384 (44.3%)	76 (27.0%)	25 (14.4%)
CT + CTA	519 (39.3%)	387 (44.7%)	89 (31.6%)	43 (24.7%)
CT + CTA + CTP	296 (22.4%)	83 (9.6%)	110 (39.0%)	103 (59.2%)
Polytrauma CT	22 (1.6%)	12 (1.4%)	7 (2.4%)	3 (1.7%)
**Diagnosis**				
Ischemic Stroke, *n* (%)	592 (44.5%)	300 (34.6%)	163 (57.8%)	129 (74.1%)
Acute Stroke	424 (71.6%)	193 (64.3%)	119 (73%)	112 (86.8%)
Media Ischemia	393 (66.4%)	168 (56.0%)	124 (76.1%)	101 (78.3%)
SVO	321 (54.2%)	228 (76%)	84 (51.5%)	9 (7%)
ICA	53 (9%)	12 (4%)	10 (6.1%)	31 (24%)
M1	88 (14.9%)	5 (1.7%)	27 (16.6%)	56 (43.4%)
M2	57 (9.6%)	19 (6.3%)	23 (14.1%)	15 (11.6%)
M3	17 (2.9%)	9 (3%)	4 (2.4%)	4 (3.1%)
VA	18 (3%)	11 (3.7%)	5 (3.1%)	2 (1.6%)
BA	9 (1.5%)	2 (0.7%)	1 (0.6%)	6 (4.7%)
P1	13 (2.2%)	8 (2.6%)	4 (2.5%)	1 (0.8%)
P2	6 (1%)	3 (1%)	3 (1.8%)	0 (0%)
A1	1 (0.2%)	0 (0%)	0 (0%	1 (0.8%)
A2	9 (1.5%)	3 (1.0%)	2 (1.2%)	4 (3.1%)
ICH, *n* (%)	110 (8.3%)	33 (3.8%)	49 (17.4%)	28 (16.1%)
Loco typico	52 (47.3%)	23 (69.7%%)	21 (42.8%)	8 (28.6%)
Atypical	58 (52.7%)	10 (30.3%)	28 (57.2%)	20 (71.4%)
SAH, *n* (%)	90 (6.8%)	65 (7.5%)	18 (6.4%)	7 (4%)
Conservative Treatment	50 (55.5%)	44 (67.7%)	4 (22.2%)	2 (28.6%)
Clipping	16 (17.8%)	9 (13.8%)	5 (27.8%)	2 (28.6%)
Coiling	24 (26.7%)	12 (18.5%)	9 (50%)	3 (42.8%)
TIA, *n* (%)	190 (14.4%)	169 (19.5%)	20 (7.1%)	1 (0.6%)
ABCD2 0–3	70 (36.8%)	67 (39.8%)	3 (15%)	0 (0%)
ABCD2 4–5	90 (47.4%)	78 (46.5%)	12 (60%)	0 (0%)
ABCD2 6–7	30 (15.8%)	23 (13.7%)	5 (25%)	1 (100%)
Mimic, *n* (%)	319 (24.1%)	289 (33.4%)	23 (8.2%)	7 (4%)
SDH, *n* (%)	21 (1.6%)	10 (1.2%)	9 (3.2%)	2 (1.1%)
Thrombectomy, *n* (%)	135 (10.2%)	15 (1.7%)	41 (14.5%)	79 (45.4%)
i.v. tPA	167 (12.6%)	48 (5.5%)	62 (22%)	57 (32.8%)
**ASPECTS**				
0–3, *n* (%)	8 (3.6%)	1 (2.1%)	1 (1.5%)	6 (5.5%)
4–5, *n* (%)	27 (12.1%)	2 (4.2%)	4 (6.1%)	21 (19.1%)
6–8, *n* (%)	61 (27.2%)	10 (20.8%)	15 (22.7%)	36 (32.7%)
9–10, *n* (%)	128 (57.1%)	35 (72.9%)	46 (69.7%)	47 (42.7%)
Previous stroke, *n* (%)	255 (19.3%)	141 (16.3%)	73 (25.9%)	41 (23.6%)
Previous TIA, *n* (%)	85 (6.4%)	61 (7.0%)	14 (5%)	10 (5.7%)
Coronary heart disease, *n* (%)	253 (19.3%)	153 (17.7%)	65 (23%)	35 (20.1%)
Peripheral artery disease, *n* (%)	71 (5.4%)	50 (5.8%)	13 (4.6%)	8 (4.6%)
Heart failure, *n* (%)	105 (7.9%)	56 (6.5%)	31 (11%)	18 (10.3%)
Smoking, *n* (%)	220 (16.6%)	151 (17.4%)	49 (17.4%)	20 (11.5%)
Alcohol, *n* (%)	83 (6.2%)	46 (5.3%)	17 (6%)	20 (11.5%)
Hypertension, *n* (%)	897 (67.9%)	562 (64.9%)	207 (73.4%)	128 (73.6%)
Hyperlipidemia, *n* (%)	507 (38.4%)	325 (37.5%)	118 (41.8%)	64 (36.8%)
Diabetes mellitus, *n* (%)	310 (23.4%)	183 (21.1%)	78 (27.7%)	49 (28.2%)
Atrial fibrillation, *n* (%)	250 (18.9%)	111 (12.8%)	67 (23.8%)	72 (41.4%)
Persistent foramen ovale, *n* (%)	359 (27.2%)	234 (27%)	84 (29.8%)	41 (23.6%)
Oral anticoagulation, *n* (%)	241 (18.2%)	138 (16.1%)	62 (22%)	41 (23.6%)
**Onset (last seen well) to door (h)**				
<4.5, *n* (%)	609 (46.1%)	337 (39%)	161 (57.1%)	111 (63.8%)
4.5–6, *n* (%)	66 (5.0%)	40 (4.6%)	16 (5.7%)	10 (5.7%)
6–12, *n* (%)	172 (13%)	113 (13%)	37 (13.1%)	22 (12.7%)
12–24, *n* (%)	278 (21%)	213 (24.6%)	42 (14.9%)	23 (13.2%)
>24, *n* (%)	197 (14.9%)	163 (18.8%)	26 (9.2%)	8 (4.6%)

*ASPECTS, Alberta Stroke Program Early CT Score; BA, basilar artery; CTA, CT angiography; CTP, CT perfusion; ICA, internal carotid artery; ICH, intraparenchymal hemorrhage; IQR, interquartile range; i.v. tPA, intravenous tissue plasminogen activator; NIHSS, National Institute of Health Stroke Scale; mRS, modified Rankin Scale; SAH, subarachnoid hemorrhage; SD, standard deviation; SDH, subdural hemorrhage; SVO, small vessel occlusion; TIA, transient ischemic attack; VA, vertebral artery*.

### Suspected Stroke Patients With Mild Symptoms (NIHSS ≤ 5)

Most patients presented with mild symptoms (866 patients; 65.5%), and among those, ischemic stroke (300 patients; 34.6%), stroke mimics (289; 33.4%), and TIA (169; 19.5%) were the most common diagnoses. Additionally, 65 patients had SAHs (7.5%), 33 (3.8%) had ICHs, and 10 (1.2%) had SDHs. Median NIHSS was 2 (IQR 2–4) and mean age was 69.1 years (SD ± 15.3); 44.3% underwent native CT only, while arterial imaging was performed in the remaining 55.7%. Eighty-three patients (9.6%) underwent additional CTP.

Out of the 300 ischemic strokes, 168 (56%) were deemed acute and a causative occlusion was found in 72 (24%) patients. Out of the 72 occlusions, 19 (26.4%) were large-vessel occlusions (defined as ICA, M1 and BA) and the remaining 53 (73.6%) were medium-vessel occlusions; for a detailed overview of the occlusion locations, please refer to Table 1.

EVT was performed in 15 patients (5%) and i.v.-tPA was administered in 48 (16%) of the ischemic strokes. ASPECTS was very low (0–3) in 1 patient (2.1%), low (4, 5) in 2 patients (4.2%), medium (6–8) in 10 patients (20.8%), and high (9, 10) in 35 patients (72.9%) with an occlusion in the anterior circulation.

Although only mildly affected, excellent functional outcome (defined as mRS ≤ 1) was only achieved in 47.1% of the patients and 70.2% were functionally independent (mRS ≤ 2) at discharge (Table 2).

**Table 2:** Overview of outcome variables stratified by severity.

**Variable**	**All patients (*n* = 1,322)**	**NIHSS ≤ 5 (*n* = 866)**	**NIHSS 6–12 (*n* = 282)**	**NIHSS ≥ 13 (*n* = 174)**
Length of stay (days), Median (IQR)	4 (1-9)	4 (1-7)	6 (1-12)	8 (3-15)
Discharge location				
Previous environment, *n* (%)	566 (42.8%)	498 (57.5%)	52 (18.4%)	16 (9.2%)
Nursing home, *n* (%)	210 (15.9%)	112 (12.9%)	62 (22.0%)	36 (21,1%)
Rehabilitation, *n* (%)	436 (33%)	225 (26%)	130 (46.1%)	81 (46.3%)
In-hospital dead, *n* (%)	112 (8.5%)	31 (3.6%)	39 (13.8%)	42 (24.1%)
NIHSS at discharge, Median (IQR)	1 (0–5)	0 (0–2)	5 (2-10)	12 (5-22)
mRS at discharge, Median (IQR)	2 (1-4)	1 (0–3)	2 (3-5)	5 (4-5)
mRS 0, *n* (%; cumulative within group %)	286 (21.6%; 21.6%)	256 (29.6%; 29.6%)	22 (7.8%; 7.8%)	8 (4.6%; 4.6%)
mRS 1, *n* (%; cumulative within group %)	271 (20.5%; 42.1%)	216 (24.9%; 54.5%)	46 (16.3%; 24.1%)	9 (5.1%; 9.7%)
mRS 2, *n* (%; cumulative within group %)	196 (14.8%; 56.9%)	144 (16.6%; 71,1%)	43 (15.2%; 39.3%)	9 (5.1%; 14.9%)
mRS 3, *n* (%; cumulative within group %)	166 (12.6%; 69.6%)	105 (12.1%; 83.2%)	44 (15.6%; 54.9%)	17 (9.7%; 24.6%)
mRS 4, *n* (%,: cumulative within group %)	159 (12%; 81.6%)	71 (8.2%; 91.4%)	54 (19.1%; 74%)	34 (19.4%; 44%)
mRS 5, *n* (%; cumulative within group %)	104 (7.9%; 89.5%)	16 (1.8%; 93.2%)	33 (11.7%; 85.7%)	55 (32%; 76%)
mRS 6, *n* (%; cumulative within group %)	112 (8.4%; 97.9%)	31 (3.6%; 96.8%)	39 (13.9%; 95.6%)	42 (24%; 100%)
mRS unknown (%; cumulative within group %)	28 (2.1%; 100%	27 (3.2%; 100%)	1 (0.4%; 100%)	0 (0%; 100%)
Hemicrani ectomy, *n* (%)	41 (3.1%)	16 (1.8%)	16 (5.7%)	9 (5.1%)

*IQR, interquartile range; NIHSS, National Institute of Health Stroke Scale; mRS, modified Rankin Scale*.

### Suspected Stroke Patients With Moderate Symptoms (NIHSS 6–12)

In this group (*n* = 282 patients; 21.3%), 163 (57.8%) patients were diagnosed as ischemic stroke, 49 (17.4%) as ICH, 18 (6.4%) as SAH, 9 (3.2%) as SDH, 20 (7.7%) as TIA, and 23 (8.2%) patients had stroke mimics. Median NIHSS was 9 (IQR 8–11) and mean age was 73.5 years (SD ± 13.8). Within this group, 27% underwent native CT only, while arterial imaging was performed in the remaining 73%. One hundred and ten patients (39%) underwent additional CTP.

Out of the 163 ischemic strokes, 124 (76.1%) were deemed acute and a causative occlusion was found in 79 (48.5%) patients. Out of the 79 occlusions, 28 (35.4%) were large-vessel occlusions and the remaining 51 (64.6%) were medium-vessel occlusions. EVT was performed in 41 patients (25.2%) and i.v.-tPA was administered in 62 (38%) of the ischemic stroke patients. ASPECTS was very low in 1 (1.5%) patient, low in 4 patients (6.1%), medium in 15 patients (22.7%), and high in 46 (69.7%) of the patients with an occlusion in the anterior circulation. Excellent outcome was achieved in 25.8%, while 39.9% were functionally independent at discharge. In-hospital mortality was 9.2%. ICHs were far more common in this group with a frequency of 17.4% (Table 2).

### Suspected Stroke Patients With Severe Symptoms (NIHSS ≥ 13)

In this group, ischemic stroke was the most common diagnosis (129 patients, 74.1%), followed by ICH (28 patients, 16.1%), SAH and stroke mimics (each 7 patients, 4%), SDH (2 patients, 1.1%), and TIA (1 patient, 0.6%). Median NIHSS was 17 (IQR 14–21) and mean age was 74.8 years (SD ± 12.7). Within this group, 14.4% underwent native CT only, while arterial imaging was performed in the remaining 85.6%. One hundred and three patients (59.2%) underwent additional CTP. Out of the 129 ischemic strokes, 101 (78.3%) were deemed acute and a causative occlusion was found in 119 (92.2%) patients. Out of the 119 occlusions, 93 (78.2%) were large-vessel occlusions and the remaining 26 (21.8%) were medium-vessel occlusions. EVT was performed in 79 (61.2%) and i.v.-tPA was administered in 57 patients (44.2%) with ischemic stroke. ASPECTS was very low in 6 (5.5%), low in 21 (19.1%), medium in 36 (32.7%), and high in 47 (42.7%) patients with an occlusion of the anterior circulation. Excellent outcome was achieved in 9.3%, while 16.3% of the patients were functionally independent at discharge. In-hospital mortality was 21.8% (Table 2).

## Discussion

Our study has several major findings: (1) it shows that a substantial proportion of patients with suspected stroke present with medium-vessel occlusions, which are potentially eligible for EVT; (2) it provides evidence that most suspected stroke patients present with mild symptoms to the emergency department (65.5% had an NIHSS ≤ 5); (3) it shows that a substantial proportion of stroke patients with an occlusion of the anterior circulation presents with an ASPECTS of ≤ 5 (15.7%); and (4) a large proportion of patients present in an extended time window of 6–24 h after onset of symptoms (34.0%) or even later than 24 h (14.9%).

These results demonstrate that it is important to plan resources in thrombectomy-capable centers not only for patients with LVO and selection criteria in randomized trials but also for patients with low NIHSS, low-ASPECTS patients, patients with distal occlusions, and patients presenting in extended time windows. Recent guidelines of the American Heart Association/American Stroke Association (AHA/ASA) and the European Stroke Organization (ESO)/European Society of Minimally Invasive Neurological Therapy (ESMINT) (8, 9) already partially account for these upcoming changes, and a recent review on expanding indication for EVT gives a foresight on the direction in which EVT will move with the help of advanced imaging and better endovascular devices (6). In this context, our study shows that an additional percentage of up to 20% of all patients with ischemic strokes might be candidates for EVT. This estimate is in line with an analysis for late-window patients by Jadhav et al. based on DAWN and DEFUSE-3 inclusion criteria (10). As we were not able to evaluate the impact of pre-stroke disability, this group might even be larger, as these patients get recognized as candidates for EVT as well (11). Therefore, resource planning in the future should address these developments since substantially higher numbers of EVT patients will lead to higher workloads for neurointerventionalists and other subspecialties such as anesthesiologists. Since the training of such specialists takes years, hospitals should act now and invest in better training capabilities (12). Moreover, our study gives detailed information about the distribution of stroke mimics such as different types of intracranial hemorrhages. It shows the overall frequency of all types of intracranial hemorrhages combined was 14.9% in our cohort which is in line with larger epidemiological studies (13, 14). It further suggests that the occurrence of ICH increases whereas SAH gets less common with increasing NIHSS.

Compared to previous studies, the frequency of LVOs was comparable with ~25% having LVOs in total, and of those, ~15% have M1, 9% have distal ICA, and 1.5% have basilar artery occlusions (5). This demonstrates the external validity of our results. The frequency of medium-vessel occlusions was with 17.4% lower compared to other studies who reported frequencies between 24 and 43% (15, 16). One possible explanation may be that only 22.4% of the patients in our study underwent perfusion imaging and medium-vessel occlusions might get frequently missed on CT angiography alone (17).

Another finding is the high morbidity and mortality in patients with mild to moderate symptoms (NIHSS ≤ 5) at presentation, with only 70% of the stroke patients being functionally independent (mRS ≤ 2) at discharge. This further underlines the potential importance of performing EVT in patients with low NIHSS. Last, our study is the first to describe the distribution of ASPECTS values in a consecutive cohort of patients with suspected stroke. This adds further information to the general understanding of the distribution of ischemic stroke patients at initial presentation and adds information on how many patients can be expected to be included when low-ASPECTS trials will present positive results for performing EVT (18, 19).

### Limitations

Our study has limitations partly attributed to its single-center, retrospective design. Moreover, more patients may have been classified as ischemic stroke and less as TIA if the admission imaging modality would have been MRI instead of CT. The definition of the upper NIHSS bound of the moderate stroke group was made based on our best judgment as there is no clear consensus on this definition and it can be argued that other values in a range from 10 to 15 would have been a better fit. However, we present a comparably large consecutive cohort with detailed clinical and imaging information that partially was not available before and that is urgently needed to adjust resources in thrombectomy-capable centers.

## Conclusion

Our results predict an increase of EVT eligible patients of up to another 20% of all ischemic stroke patients. In this context, this study helps to plan resources in thrombectomy-capable centers in times of expanding indications for EVT where resources will have to be adjusted to patients with low-NIHSS, low-ASPECTS, distal occlusions, and patients presenting in the extended time window.

## Data Availability Statement

The raw data supporting the conclusions of this article will be made available by the authors, without undue reservation.

## Author Contributions

AB, NN, IT, and CH designed the data collection sheets, collected the data, and performed the analysis. PS, AB, and MP wrote the manuscript. All authors gave final approval of the submitted version and agreed to be accountable for all aspects of the work in ensuring that questions related to the accuracy or integrity of any part of the work are appropriately investigated and resolved and critically reviewed the manuscript.

## Conflict of Interest

The authors declare that the research was conducted in the absence of any commercial or financial relationships that could be construed as a potential conflict of interest. The reviewer, PR, declared a past co-authorship with three of the authors, PS, AB, and MP, to the handling editor. The handling editor declared a past collaboration with the authors.

## Publisher's Note

All claims expressed in this article are solely those of the authors and do not necessarily represent those of their affiliated organizations, or those of the publisher, the editors and the reviewers. Any product that may be evaluated in this article, or claim that may be made by its manufacturer, is not guaranteed or endorsed by the publisher.
